# Effect of *Pinus taeda* Hydrolyzed Lignin on Biochemical Profile, Oxidative Status, and Semen Quality of Healthy Dogs

**DOI:** 10.3389/fvets.2022.866112

**Published:** 2022-06-01

**Authors:** Giulio G. Aiudi, Vincenzo Cicirelli, Aristide Maggiolino, Matteo Burgio, Andrea Bragaglio, Alessandra Tateo, Pasquale De Palo

**Affiliations:** Department of Veterinary Medicine, University of Bari A. Moro, Bari, Italy

**Keywords:** antioxidants, semen quality, oxidative status, dogs, polyphenols

## Abstract

Sub-fertility represents a frequent challenge in canine reproduction. The use of micronutrients and/or additives was investigated as an approach to improve sperm quality, which are the main constraints on reproduction in canine species. Although some information is available about the effect of daily supplementation with substances presenting antioxidant/antioxidative activity on semen quality, this study aimed to observe the effect of a polyphenolic mix of substances derived from hydroxylation of *Pinus taeda* lignin (PTHL). For the trial, 40 male dogs were involved, 20 received PTHL for 90 days and 20 were left untreated, serving as a control group. Every 30 days, blood and semen samples were collected and analyzed. The biochemical profile of both groups was not affected by treatment and time (*p* > 0.05). Differently, dogs that received PTHL showed higher blood superoxide dismutase (SOD), catalase (CAT), and glutathione peroxidase (GPx) activity (*p* < 0.01). Moreover, the dietary addition of PTHL can significantly increase the semen volume, concentration, and spermatozoa motility (*p* < 0.01) in healthy dogs. PTHL supplementation represents a good way to enhance the semen quality of dogs and improve the antioxidant status of animals.

## Introduction

Several clinical studies suggested that, among nutritional factors, fish-derived n-3 polyunsaturated fatty acids (PUFAs) may exert a positive effect on sperm motility and fertility. Since several PUFAs are essential in many animals, their requirements must be covered by dietary intake (1, 2). Other microelements, such as selenium, copper, and zinc, act directly or indirectly on sperm metabolism (3, 4). Many researchers have shown that nutritional deficiencies can lead to reduced sperm quality through defective spermatogenesis or by generating intense oxidative stress (5). Oxidative damage can cause sperm dysfunction, such as loss of motility and vitality (6, 7). Therefore, antioxidants may play a key role by protecting male stem cells from oxidative damage (8) and by preventing loss of motility and impaired sperm-oocyte fusion capacity (9). However, since the development of canine artificial insemination with frozen semen, various known substances with antioxidant activity, such as vitamins E and C, glutathione, and butylated hydroxytoluene, have been added to the cryopreservation extenders (10–14). Nevertheless, few studies have been conducted on the potential effects of some dietary antioxidant substances on dogs' semen quality. Several vegetable matrices are rich in bioactive compounds, such as phenolic compounds or tannins with high antioxidant activity, and this makes them potentially suitable for animal feeding (15). Several studies have highlighted the benefits of consuming extracts and foods rich in these compounds (16). Although the mechanisms are unclear, polyphenols and tannins improve the antioxidant status of cells and tissues in humans (17), rats (18), mice (19), livestock (20, 21), and pets (22, 23). One of these natural substances is Pinus taeda hydrolyzed lignin (PTHL), derived from P. taeda, commonly known as loblolly pine, a very common species of tree in Northern America. Lignin is often an agricultural by-product, and is very difficult to be recycled, although it is organic matter. The possibility of a circular economy model where lignin is converted into high added value products also represents an interesting focus from an environmental sustainability perspective. The PTHL is a polyphenol mixture derived from these trees' lignin that proved to have a positive effect on animal welfare (24). The present study aims to evaluate the effect of PTHL oral administration on dogs' plasma oxidative status, biochemical profile, and semen characteristics.

## Materials and Methods

The protocol for animal research was approved by the Ethics Committee for animal testing–CESA (656/18 – III/13) of the Department of Veterinary Medicine of the University of Bari “Aldo Moro,” Bari, Italy.

### Animals

**Figure 1:**
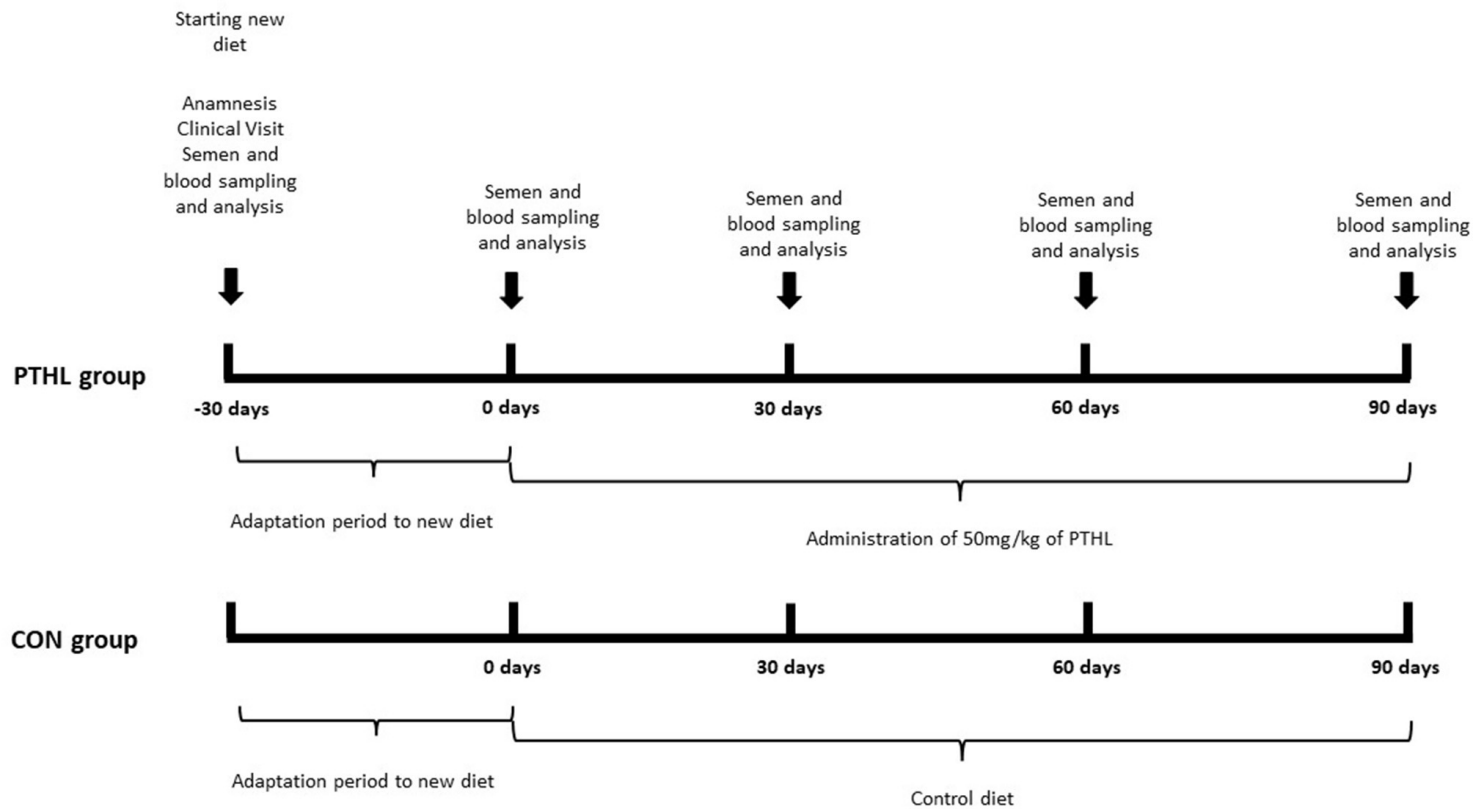
Experimental design. PTHL, Pinus taeda hydrolyzed lignin; CON, Control.

The study was performed during the spring of 2021 at the Obstetric, Gynecological, and Andrological Clinic of the Veterinary Hospital of the Veterinary Medicine Department of the “Aldo Moro” University of Bari (Italy). Dogs' experimental management is reported in Figure 1. The dogs were clinically examined to ensure their health status 30 days before the commencement of the trial. Each dog has submitted for a clinical examination and blood analyses (hematocrit and total protein) (25, 26). An ultrasound exam of the reproductive tract was also performed, and it was verified that all dogs reacted positively to sperm collection by digital manipulation. Only normospermic male pet dogs kept in the house were included. In the end, forty mixed-breed dogs were included in the present study (Supplementary Table S1 reported all animals characteristics). Dogs were healthy with a mean age of 3.8 years, a mean body weight of about 27 kg, and a mean body condition score (BCS) of 3. All the dogs started to be fed with the same commercial feed [10 g/kg of body weight (BW) daily, composition reported in Table 1] two times a day to ensure a 30-days period of adaptation to the new commercial feed before starting the trial.

**Table 1:** Composition of diet commercial feed.

**Item**	**On 100 g of product**
Moisture	10 g
Raw protein	28 g
Fat	15 g
Raw fibers	3 g
Ashes	9 g
Vitamin A	1,500 IU
Vitamin D	100 IU
Vitamin E acetate (alpha-tocopherol 91%)	12.5 mg
Vitamin B2	2.6 mg
Vitamin B6 (pyridoxine hydrocloride)	0.5 mg
Vitamin B1 (thiamine mononitrate)	0.6 mg
Choline chloride	75 mg
Iodine (anhydrous calcium iodate)	0.075 mg
D-panthotenic acid	1 mg
Vitamin H (Biotin D)	0.05 mg
Calcium	0.5 g
Vitamin K3 (menadione)	0.125 mg
Vitamin PP (nicotine acid)	2.5 mg
Vitamin B12	0.0035 mg
Folic acid	0.1 mg
Cobalt (basic cobalt carbonate)	0.015 mg
Iron (ferrous carbonate)	2 mg
Manganese (manganous oxide)	4 mg
Copper (Copper sulfate, pentahydrate)	1 mg
Selenium (sodium selenite)	0.01 mg
Zinc (zinc oxide)	3 mg

### Experimental Protocol

The dogs were randomly assigned to one of two equal-sized groups (*n* = 20) using www.randomizer.org (27) (Figure 1).

One group was the experimental one (PTHL) and the other was the control group (CON). The PTHL group received the supplement containing PTHL (Oxilem®, I-Green, Padua, Italy). Table 2 reports the chemical composition and antioxidant activity of PTHL (24). The PTHL group received 500 mg/kg of PTHL each day during the 90 days of the trial. The dose was orally administered in powder according to the manufacturer's instructions, based on empirical trials (IGreen, Padua, Italy). The supplement was orally administered. During the trial, semen and blood samples collection were performed before starting the trials (day 0) and 30, 60, and 90 days.

**Table 2:** Phenolic composition and antioxidant activity of *Pinus Taeda* hydrolyzed lignin (PTHL)^a^.

**Item**	
**Determined composition (g/100 g)**	
Vanillin	26.4
Eriodictyol	3.4
Quercetin	2.7
Isorhamnetin	1.6
Rosmarinic acid	1.4
Quercetin ramnoside	13.9
Methylgallate retunoside	42.3
Epigallocatechin-3-methylgallate	1.5
Ferulic acid derivates	6.7
**Antioxidant activity (μmol TE^b^ g^−1^ DW^c^)**	
Trolox equivalent antioxidant capacity	23.9
Oxygen radical absorbance capacity	122.4

a *According to Gerardi et al. (28) and Blando et al. (29)*.

b *Trolox equivalents*.

c *Dry weight*.

−1 *is the measure unit for those parameters calculated on g elevated at ^−1^*.

### Blood Samples and Analysis

Blood was aseptically collected *via* cephalic vein puncture using disposable needles (22G), with a negative pressure 4 ml tube system for serum (without anticoagulant) and plasma (with 15 USP U/ml of heparin) (Becton, Dickinson Canada Inc, Vacutainer 1, Oakville, Canada). Tubes for plasma were immediately centrifuged (1,500 × g for 10 min) while tubes for serum were allowed to clot at a refrigerated temperature for 10 min prior to being centrifuged (1,500 × g for 10 min). All plasma and serum aliquots were stored at −80°C until analyses. Clinical biochemistry parameters were obtained from the serum samples using an automated biochemistry analyzer (CS-300B; Dirui, Changchun, China) as described by De Palo et al. (30). The following parameters were analyzed: alanine aminotransferase (ALT), aspartate aminotransferase (AST), creatine phosphokinase (CPK), lactate dehydrogenase (LDH), alkaline phosphatase (ALP), glucose (Glu), blood urea nitrogen (BUN), creatinine (Crea), total serum protein (TP), albumin (Alb), cholesterol (Chol), triglycerides (Trig), non-esterified fatty acids (NEFA), calcium (Ca), phosphate (P), magnesium (Mg), chloride (Cl), (Gesan Production Kit, Campobello di Mazara, Trapani, Italy). Besides, globulins (Glob) and albumin/globulin ratio (Alb/Glob) were calculated starting from total protein and albumin parameters. Standard assay kits were used to calibrate the multi-parameter analyzer (Seracal, Gesan Production Kit, Campobello di Mazara, Trapani, Italy) before each analytical session. After setting the calibration curve, two multi-parameter control sera, a normal and a pathological one (Seracontrol N and Seracontrol P, Gesan Production Kit, Campobello di Mazara, Trapani, Italy), were used to verify internal accuracy, considered satisfactory when the measured value deviated by no more than 3.00% from the manufacturer's declared values. Each sample was analyzed in triplicate, and the value used in the raw dataset was the arithmetic mean of the three recordings for each item. Plasma samples were used for oxidation parameters and antioxidant enzyme activities assays. Thiobarbituric acid reactive substances (TBARS) were determined spectrophotometrically as described by Maggiolino et al. (31), by adding 100 ml of plasma to a 3.7 μl/ml thiobarbituric acid solution. Plasma reactive carbonyl derivative (RCD) levels were determined according to Faure and Lafond (32) using the carbonyl reagent DNPH. Plasma (200 ml) was mixed with 1 ml of water and 2 ml of 200 μl/ml trichloroacetic acid and centrifuged at 1,000 × g for 10 min. The pellet was resuspended in 1 ml of 10 mmol/L DNPH and incubated for 60 min at 37.8°C. For control, 1 ml of 1 mol/L hydrochloric acid was used instead of DNPH. Subsequently, 1 ml of 200 μl/ml trichloroacetic acid was added, and the sample was centrifuged at 1,000 × g for 10 min. The pellet was washed with 1:1 ethanol-ethyl acetate solution and centrifuged at 1,000 × g for 10 min. The pellet was mixed with 1 ml of 6 mol/L guanidine (diluted in 20 mmol/L dihydrogenphosphate at pH 2.3). Finally, the sample was incubated for 40 min at 37.8°C. The absorbance was measured at 380 nm.

Hydroperoxides (Hy) were determined spectrophotometrically by an iodometric method as described by Maggiolino et al. (33). Aliquots (90 ml) of plasma were put into eight microcentrifuge vials (1.5 ml). Then, 10 μl of 10 mM TPP in methanol were added to four of the vials to reduce ROOHs, thereby generating a quadruplicate of blanks. Methanol (10 ml) was added to the remaining four vials to produce a quadruplicate of test samples. All the vials were then vortexed and incubated at room temperature for 30 min prior to the addition of 900 ml of FOX2 reagent. After mixing, the samples were incubated at room temperature for 30 min. The vials were centrifuged at 2,400 × g for 10 min with a swing-out rotor (Hettich Rotenta/RP centrifuge, Hettich-Zentrifugen, Tuttlingen, Germany). The absorbance of the supernatant was measured at 560 nm using an Ultraspec 2000 spectrophotometer (Pharmacia Biotech, Uppsala, Sweden). The ROOH concentration in the plasma samples was calculated using the mean absorbance difference between quadruplicates of test samples and blank samples.

Protein carbonyls (PC) levels were determined spectrophotometrically as reported by Salzano et al. (21). The superoxide dismutase (SOD) (EC 1.15.1.1) activity was examined according to Misra (34), and the enzymatic activity was based on the 50% inhibition rate of epinephrine auto-oxidation at 480 nm (35). The SOD activity was assessed as the 50% inhibition rate of epinephrine auto-oxidation at 480 nm. The epinephrine autoxidation stimulation by traces of heavy metals present as contaminants in the reagents was prevented by adding 10^−4^ *M* EDTA in the buffer to chelate those ions. The SOD activity was expresses as U/ml.

The catalase (CAT) (EC 1.11.1.6) activity was assayed by the method of Clairborne (36) as described by Tateo et al. (37). The amount of enzyme required to degrade 1 μmol of H_2_O_2_ in 60 s was defined as 1 unit of enzyme activity by following the decrease in absorbance of H_2_O_2_ at 240 nm (e = 40 M^−1^ cm^−1^). Its activity was expressed as U/mg of protein.

The glutathione peroxidase (GPx) (EC1.11.1.9.) activity was measured according to Gunzler (38) as described by Dinardo et al. (39). The analysis was based on the measure of the rate of reduced glutathione oxidation by tertbutyl hydroperoxide, catalyzed by GPx. The constant concentration of reduced glutathione was ensured by the addition of exogenous glutathione reductase and NADPH, which converted the oxidized glutathione to reduced glutathione. The rate of oxidized glutathione formation was then measured by the change in the absorbance of NADPH at 340 nm. Its activity was expressed as nmol of NADPH oxidized/min per ml.

### Semen Collection and Computer Assisted Sperm Analysis

Dog's semen was collected into an artificial vagina by manual stimulation, while the dogs sniffed swabs of bitches in estrous (using natural estral pheromones). The ejaculate collection was performed using 3 different Falcon tubes, one for each part of the semen: urethral, spermatic, and prostatic (40, 41). The ejaculation analysis was performed as described by Alonge et al. (42). The second seminal part was analyzed by the Computer Assisted Sperm Analyzer (43) (CASA, IVOS-Sperm CASA system, Version 12.3, Hamilton Thorne, MA, USA). The CASA software (IVOS 12.3 version) was set up for canine semen-speficic parameters as reported in Table 3. According to the manufacturer's instructions, for each analysis, a 3 μl drop from each sperm sample was diluted 5 times in Tris-Fructose extender and put on a Leja slide 4 chambers of 20 μm (Leja Products B.V. Nieuw Vennep, The Netherlands). The Leja slide was positioned in the dedicated chamber of the microscope, allowing it to settle for a few seconds before analysis. The computerized analyzer scanned five random non-consecutive microscopic fields. The parameters evaluated were: ejaculate volume, concentration, total motility, and percentage of motile spermatozoa (progressive motility), velocity average pathway (VAP), straight-line velocity (VLS), curvilinear velocity (VCL), amplitude of lateral head displacement (ALH), beat-cross frequency (BCF), straightness (STR), linearity (LIN), and total number of counted cells (TSC). VAP was elaborated by the software as the average velocity of smoothed cell path, expressed in μm/s. Then, the overall sperm population was divided into 4 groups based on the velocity, according to low VAP cut-off (LVV) and medium VAP cut-off (MVV). Thus, sperms were classified as follows: rapid spermatozoa, with VAP > MVV; medium spermatozoa, with LVV < VAP < MVV; slow spermatozoa, with VAP < LVV; and static spermatozoa, represented by the fraction of those cells not moving during the analysis (44).

**Table 3:** The IVOS version 12.3 software settings for dog semen parameters.

**Parameters**	**Cut-off value**
Frames per second (Fps)	30
Frequency	60 Hz
Temperature of analysis	37°C
Minimum contrast	75
Minimum cell size	4 pixels
Progressive cell cut-off	100 μm/s; 75% STR
Low VAP cut-off	9 μm/s
Low VSL cut-off	20 μm/s

### Statistical Analysis

The dataset was tested for normal distribution and variance homogeneity (Shapiro–Wilk test). Afterward, all data were subjected to analysis of variance (ANOVA) using the general linear model (GLM) procedure as reported the following model:


$$\begin{array}{l} Y_{\text{ijkl}}=\text{μ}+\alpha_{\text{i}}+O_{\text{j}}+T_{\text{k}}+{\left(N\times T\right)}_{\text{jk}}+\;{\text{ε}}_{\text{ijkl}} \end{array}$$

where Y_ijkl_ represents the dependent variables, μ is the overall mean; α_i_ is the ith dog random effect (i = 1,…40), O_j_ is the effect of the jth oral administration treatment (j = 1, 2), T_k_ is the effect of the kth time (1,…4), (O × T)_jk_ is the binary interaction effect of jkth (1,…8) treatment × time and ε_ijk_ is the error term. A Tukey test for repeated measures with respect to time was applied to evaluate the differences among means when the effect of time or the binary interaction of treatment × time was significant. The significance level was set at *p* < 0.05, and the results were expressed as means and mean standard error (SE). Data analysis was performed using SAS software (46).

## Results

Tables 4, 5 show serum biochemical-clinical and electrolyte profile results between the two experimental groups (PTHL vs. CON) during the trial. No differences were observed according to time, PTHL inclusion, and their binary interaction (*p* > 0.05).

**Table 4:** Total protein, albumin, urea, uric acid, creatinine, bilirubin, triglyceride, glycemia, alanine amino transferase (ALT), aspartate amino transferase (AST), alkaline phosphatase (ALP), and cholesterol serum concentration in dogs treated with dietary supplementation of PTHL and untreated dogs (CON) for 90 days.

**Parameter**	**Group**	**Time (days)**				**SEM^a^**	***p*** **-value**			**Reference value^c^**
		**0**	**30**	**60**	**90**		**Group**	**Time**	**G × T^b^**	
Total protein (g/dL)	CON	6.67	6.55	7.00	6.72	0.325	0.049	0.904	0.591	5.4–7.1
	PTHL	6.38	6.10	6.12	6.16					
Albumin (g/dL)	CON	2.98	2.95	3.05	2.95	0.105	0.671	0.748	0.985	2.6–3.3
	PTHL	3.02	2.94	2.98	2.90					
Urea (mmol/L)	CON	59.26	58.95	53.70	60.10	9.890	0.459	0.290	0.919	21–60
	PTHL	52.26	51.02	55.02	57.44					
Uric acid (mg/dL)	CON	0.81	0.92	1.20	1.15	0.190	0.036	0.202	0.534	0.0–2.0
	PTHL	0.62	0.58	0.64	0.86					
Creatinine (mg/dL)	CON	1.42	1.50	1.42	1.42	0.100	0.047	0.473	0.529	0.5–1.5
	PTHL	1.32	1.28	1.34	1.32					
Bilirubine (mg/dL)	CON	0.26	0.40	0.47	0.42	0.135	0.016	0.135	0.426	0.1–0.5
	PTHL	0.22	0.18	0.24	0.38					
Triglyceride (mg/dL)	CON	36.98	32.40	32.57	31.30	9.105	0.337	0.179	0.542	30.1–38.1
	PTHL	38.57	38.62	35.60	37.26					
Glycemia (mg/dL)	CON	86.96	61.20	81.60	71.70	6.100	0.008	0.174	0.163	65–118
	PTHL	89.43	86.74	87.04	88.70					
ALT^d^ (IU/L)	CON	31.82	38.50	53.30	36.75	5.750	0.335	0.154	0.230	21–102
	PTHL	34.65	43.20	34.96	34.64					
AST^e^ (IU/L)	CON	24.78	27.47	27.15	28.30	2.215	0.442	0.511	0.241	0.0–66.0
	PTHL	21.74	23.86	22.00	23.76					
ALP^f^ (IU/L)	CON	22.50	19.05	23.40	16.52	5.115	0.178	0.932	0.649	20–156
	PTHL	23.39	23.78	26.04	28.86					
Cholesterol (mg/dL)	CON	172.46	182.90	206.67	201.40	18.650	0.089	0.374	0.747	135–270
	PTHL	163.06	153.42	168.78	177.32					

a *Standard error of the means*.

b *Group × Time*.

c *Kaneko et al. (45)*.

d *Alanine aminotransferase*.

e *Aspartate aminotransferase*.

f *Alkaline phosphatase*.

**Table 5:** Chlorine, sodium, potassium, magnesium, phosphorus, and calcium serum concentration in dogs treated with dietary supplementation of PTHL and untreated dogs (CON) for 90 days.

**Parameter**	**Group**	**Time (days)**				**SEM^a^**	***p*** **-value**			**Reference value^c^**
		**0**	**30**	**60**	**90**		**Group**	**Time**	**G × T^b^**	
Chlorine (mEq/L)	CON	112.82	112.62	114.40	110.17	2.850	0.502	0.334	0.653	105.0–115.0
	PTHL	115.14	112.52	113.30	114.90					
Sodium (mmol/L)	CON	150.15	155.22	151.42	147.95	3.035	0.421	0.935	0.199	141.0–152.0
	PTHL	149.68	145.38	151.16	151.04					
Potassium (mg/dL)	CON	4.46	4.44	4.49	4.49	0.135	0.041	0.622	0.659	4.4–5.3
	PTHL	4.19	4.16	4.20	4.22					
Magnesium (mg/dL)	CON	2.08	1.90	2.25	2.25	0.435	0.640	0.065	0.945	1.8–2.4
	PTHL	2.22	1.98	2.20	2.40					
Phosphorus (mg/dL)	CON	4.24	4.70	5.05	3.72	0.425	0.471	0.297	0.447	2.6–6.2
	PTHL	3.96	4.20	4.20	4.20					
Calcium (mg/dL)	CON	10.45	10.40	10.67	10.12	0.340	0.246	0.791	0.791	9–12
	PTHL	10.26	9.88	10.14	10.04					

a *Standard error of the means*.

b *Group × Time*.

c *Kaneko et al. (45)*.

Results for oxidation metabolites and antioxidant enzymes are reported in Table 6. Metabolites derived from lipid (TBARS and Hy) and protein (protein carbonyls) oxidation did not vary over time and between groups (*p* > *0.05)*. The ferric reducing ability of plasma (FRAP) showed higher values in PTHL groups after 90 days of trial (*p* < *0.01*). Observing enzyme activity in the PTHL group, SOD activity constantly increased during the trial (*p* < 0.01), the CAT and GPx activity increased in the first 30 days (*p* < 0.01) and then remained constant. These enzymes did not show any difference in the CON group (*p* > 0.05) during the trial. Moreover, from 30 days to the end of the trial, all these enzymes showed higher values in the PTHL group compared with the CON one (*p* < 0.01).

**Table 6:** Thiobarbituric acid reactive substances (TBARs), hydroperoxides (Hy), protein carbonyls, ferric reducing antioxidant power (FRAP), superoxide dismutase (SOD), catalase (CAT), and glutathione peroxidase (GPx) serum concentration in dog treated with dietary supplementation of PTHL and untreated dog (CON) for 90 days.

**Parameter**	**Group**	**Time (days)**				**SEM^1^**	***p*** **-value**		
		**0**	**30**	**60**	**90**		**Group**	**Time**	**G × T^2^**
TBARs (mmol/mL)	CON	1.14	1.11	1.14	1.02	0.105	0.223	0.042	0.622
	PTHL	1.39	1.37	1.16	1.13				
Hydroperoxides (mmol/mL)	CON	5.18	5.27	5.75	4.53	0.495	0.554	0.307	0.836
	PTHL	4.93	5.33	5.76	5.31				
Protein carbonyls (μmol/mg Protein)	CON	98.95	91.26	92.06	93.47	2.630	0.050	0.192	0.667
	PTHL	96.04	96.76	96.04	99.00				
FRAP (μmol TE/mL)	CON	48.40	46.96	46.77	44.55^X^	0.380	<0.0001	<0.0001	<0.0001
	PTHL	49.44	48.05	49.81	51.10^Y^				
SOD (U/mL)	CON	48.41^A^	49.84^X^	54.52^X^	54.07^X^	1.140	0.522	<0.0001	<0.0001
	PTHL	20.39^A^	60.05^BY^	72.40^CY^	91.91^DY^				
CAT (U/mL)	CON	2.88^A^	3.21^X^	3.33^X^	3.28^X^	0.125	0.320	<0.0001	<0.0001
	PTHL	2.12^A^	4.30^BY^	4.24^BY^	4.34^BY^				
GSPx (nmol NADPH ox/mL)	CON	2.43^A^	2.43^X^	2.52^X^	2.63^X^	0.035	0.106	<0.0001	<0.0001
	PTHL	2.27^A^	2.91^BY^	2.96^BY^	3.27^BY^				

1 *Standard error of the means*.

2 *Group × Time*.

*Different letters on the same line show statistical differences during time in the same group: A, B, C, D = p < 0.01; Different letters on the same row show statistical differences between groups at the same aging time: X, Y = p < 0.01*.

Table 7 shows semen volume, concentration, VAP, VLS, VCL, ALH, BCF, STR, and LIN results. Not all of them showed variation during the trial in the CON group (*p* > 0.05). Semen volume (*p* < 0.05) and concentration (*p* < 0.01) increased in PTHL dogs during the trial, with higher values in PTHL animals compared with the CON group (*p* < 0.01). The VSL, BCF, LIN (*p* < 0.01), VCL, ALH, and STR (*p* < 0.05) values increased after 90 days of PTHL administration. Only VSL and LIN showed higher values compared with CON dogs after 60 days (*p* < 0.01).

**Table 7:** Volume, concentration, velocity average pathway (VAP), straight line velocity (VLS), curvilinear velocity (VCL), amplitude of lateral head displacement (ALH), beat-cross frequency (BCF), straightness (STR), and linearity (LIN) of semen in dog treated with dietary supplementation of PTHL and untreated dog (CON) for 90 days.

**Parameter**	**Group**	**Time (days)**				**SEM^1^**	***p*** **-value**		
		**0**	**30**	**60**	**90**		**Group**	**Time**	**G × T^2^**
Volume (mL)	CON	4.86	3.65^X^	3.60^X^	4.25^X^	1.770	0.005	0.045	0.284
	PTHL	4.78^a^	6.80^bY^	6.18^bY^	7.06^bY^				
Concentration (M/mL)	CON	122.86	109.55	120.32^x^	121.47^x^	28.805	0.023	0.001	0.163
	PTHL	121.96^A^	157.56^A^	191.14^ABy^	239.44^By^				
VAP (μm/s)	CON	103.20	106.72	105.67	106.62	9.140	0.023	0.203	0.336
	PTHL	102.96	114.88	125.66	136.70				
VSL (μm/s)	CON	78.60	81.72	83.55^X^	84.87^X^	5.755	0.001	0.012	0.143
	PTHL	80.40^A^	93.04^AB^	103.52^ABY^	110.14^BY^				
VCL (μm/s)	CON	141.98	143.32	145.62	152.67	9.825	0.009	0.055	0.353
	PTHL	143.00^a^	160.24^ab^	171.98^ab^	186.22^b^				
ALH (μ)	CON	5.24	5.87	5.67	5.80	0.380	0.018	0.016	0.301
	PTHL	5.57^a^	6.22^ab^	6.68^ab^	7.10^b^				
BCF (Hz)	CON	20.50	22.32	23.65	23.95	1.290	0.044	0.000	0.207
	PTHL	21.05^A^	23.40^ABa^	24.92^ABb^	29.20^B^				
STR (VSL/VAP)	CON	79.80	32.50	80.75	83.00	5.780	0.011	0.036	0.296
	PTHL	80.78^a^	85.20^ab^	88.20^ab^	90.00^b^				
LIN (VSL/VCL)	CON	53.80	54.00	56.00^X^	56.00^X^	1.755	<0.0001	<0.0001	0.020
	PTHL	54.44^A^	57.60^ABa^	65.40^ABbY^	65.80^BY^				

1 *Standard error of the means*.

2 *Group × Time*.

*Different letters on the same line show statistical differences during time in the same group: A, B = p < 0.01; a, b = p < 0.05; Different letters on the same row show statistical differences between groups at the same aging time: X, Y = p < 0.01; x, y = p < 0.05*.

Spermatozoa motility results are reported in Table 8. Rapid movements increased after 30 days in PTHL dogs (*p* < 0.05), but no differences between groups were observed (*p* > 0.05). Slow and static movements decreased (*p* < 0.01) after 30 days of PTHL assumption, while no variations were observed in the CON group (*p* > 0.05), with lower values in PTHL dogs compared with CON ones (*p* < 0.01).

**Table 8:** Total motility, progressive motility, rapid, medium, slow, and static movements of spermatozoa in dog treated with dietary supplementation of PTHL and untreated dog (CON) for 90 days.

**Parameter**	**Group**	**Time (days)**				**SEM^1^**	***p*** **-value**		
		**0**	**30**	**60**	**90**		**Group**	**Time**	**G × T^2^**
Total motility (%)	CON	73.80	75.02	77.62	76.07	3.750	0.015	0.112	0.306
	PTHL	76.21	81.40	92.80	96.00				
Progressive motility (%)	CON	54.80	58.07	60.67	59.85	4.365	0.007	0.012	0.200
	PTHL	57.44	64.00	71.20	78.20				
Rapid (%)	CON	58.60	60.00	63.00	62.00	2.245	0.002	0.050	0.242
	PTHL	59.89^a^	72.80^ab^	79.20^ab^	82.00^b^				
Medium (%)	CON	10.00	14.00	12.00	13.00	2.445	0.209	0.462	0.786
	PTHL	12.20	13.00	8.40	8.80				
Slow (%)	CON	18.20	11.50	11.75^X^	13.00^X^	2.775	0.005	0.002	0.377
	PTHL	16.58^A^	7.80^AB^	6.80^ABY^	3.40^BY^				
Static (%)	CON	13.20	12.75^X^	13.25^X^	11.75^X^	2.565	0.009	0.037	0.378
	PTHL	12.04^A^	6.40^BY^	6.40^BY^	5.80^BY^				

1 *Standard error of the means*.

2 *Group × Time*.

*Different letters on the same line show statistical differences during time in the same group: A, B = p < 0.01; a, b = p < 0.05; different letters on the same row show statistical differences between groups at the same aging time: X, Y = p < 0.01; x, y = p < 0.05*.

## Discussion

### Blood Metabolites and Antioxidant Status

The overall data obtained from the serum biochemical and electrolyte profiles in dogs of both experimental and control groups showed ranges almost comparable with those considered physiological for this species (47, 48). The diet inclusion of PTHL in dogs did not affect the dogs health, considering that all blood constituents alteration makes it possible to hypothesize and potentially diagnose organs functionality alterations (49). For example, ALT activity is strongly related to liver function, AST to muscle and liver cell function (50), and ALP has a widespread tissue distribution, with a lot of isoenzymes isolated from other different tissues, such as liver, bone, intestinal mucosa, kidney, and leucocytes, but all of them showed values within ranges reported in the literature (51), demonstrating that treatment did not cause any tissue damages. Animals' antioxidant defense can be raised *in vivo* (enzymatic) or can derive from the diet (non-enzymatic) (52). For this, TBARs, Hy, and protein carbonyls are considered biomarkers of cell damage: their increase can be generated by oxidative stress and a boost of oxygen reactive substances (ROS). Additionally, FRAP values allow an overall evaluation of plasma antioxidant capacity (53, 54). It is well known that the dietary assumption of high amounts of substances with antioxidant activity results in the transfer of these molecules to different animal tissues, usually followed by a significant increase in their total antioxidant capacity (55), and that several polyphenolic molecules in plants possess antioxidant activities (23). Research is raising interest in these compounds due to their beneficial effects on health as anticarcinogenic (56), anti-inflammatory (57), immune and microbial modulating (58, 59), especially if taken through the diet. Several studies confirm a positive correlation between total antioxidant activity and the total phenol content assumed with diet, for example, spinach and broccoli (60, 61). However, the pharmacokinetics and antioxidant activity of polyphenols are still unclear in dogs (48, 62). Generalized stress induces a systemic increase in oxidation products.

The patterns that do not foresee the activation of the endogenous antioxidant systems, and specifically the catabolites of lipid and protein oxidation (TBARS, hydroperoxides, and protein carbonyls) measured in the plasma, did not show any difference between the two groups of animals during the entire experimental test. However, this does not exclude the hypothesis that the inclusion of PTHL in the diet of dogs with different stressful conditions (work, running, specific pathologies, etc.) may induce a variation in the production of these metabolites. On the other hand, the maximum antioxidant capacity measured by laboratory techniques is different, i.e., the animals that have taken PTHL show greater enzymatic activity and higher levels of FRAP. These results may indicate a potential positive effect of PTHL dietary supplementation against cellular oxidative stress and we can hypothesize that animal under different conditions, i.e., living stress challenges, could show greater differences on these parameters after PTHL assumption. In fact, the enzymatic activity evaluated through the used techniques highlights a capacity to react to an *in vitro* insult and therefore potential greater antioxidant response. These results agree with what observed by other authors studying the inclusion of polyphenols in the diet (62). On the other hand, increased enzymes activity represented a positive result for dog health considering that these enzymes serve as an endogenous defense against oxidative stress phenomena by determining free radical neutralization (63).

### Semen Quality and Motility

Infertility is a common problem in the reproduction of dogs. For this reason, several authors suggested different food supplements to improve the quality of the ejaculate (64). According to recent studies (5, 42), the dietary approach by balanced food integration is an effective method for the improvement of fertility in dogs. Present results showed enhancing effects of a 2-month natural antioxidant diet supplementation on canine spermatozoa, as already observed in dogs' sperm quality (42), as well as in other species (65) by other authors. Specifically, the integration of PTHL can significantly increase the semen volume and spermatozoa motility in healthy normospermic dogs. In fact, after only 30 days, the PTHL group generally showed considerable improvements from the feed integration. Furthermore, in the PTHL group, the progressive motility and the percentage of rapid-movement sperms significantly increased, while the percentage of slow and static sperms significantly decreased. Indeed, thanks to PTHL feed supplementation, the sperm quality was significantly improved in all dogs in the treated group. Although the first evidence is evaluable after 30 days of supplementation, the best results have been reached at 60 days of administration, which approximately corresponds to the physiological length of the total duration of spermatogenesis in dogs (61.9 ± 0.14 days; (66)). The improvements in sperm motility depend probably on the antioxidant power of polyphenols contained in PTHL, which is able to improve sperm quality at the beginning of the sperm differentiation and development. It is well known that oxidative stress is one of the major issues associated with sperm function (67, 68) and sperm motility is one of the factors limiting male fertility (69). Although all dogs enrolled were healthy without problems of fertility, the PTHL group showed higher total sperm count, concentration, and progressive motility, compared with the CON group. Moreover, the integration of antioxidants, compared with the normal diet, is associated with a higher number and better motility of spermatozoa (70). In fact, spermatozoa are sensitive to oxidative stress because they are well endowed with polyunsaturated fatty acids, which are vulnerable to free radical attack at the alpha methylene carbons adjacent to the carbon–carbon double bonds (6). In addition, the spermatozoa's capacity for antioxidative defense is relatively low/vulnerable in comparison to other cells/tissues that are susceptible (71). Furthermore, spermatozoa are attacked by the oxidative effect of leukocytes, such as neutrophils, which are present in the male genitals for infection and other causes (67). The decrease in concentration, motility, and function of canine spermatozoa in dogs can be attributed to poor food intake, reduced absorption, increased losses, or augmented demand for microelements. Recent literature in farm animals show that natural polyphenols are used to reduce oxidative stress because of their antioxidant properties (15, 21, 72, 73). The results of our study are biologically plausible. The production of reactive oxygen species (ROS) causes loss of motility and a decreased capacity for sperm–oocyte fusion, inducing harmful chemical and structural changes to sperm DNA, proteins, and lipids of plasma and mitochondrial membranes (9). ROS induces changes in the sperm membrane altering its fluidity, resulting in loss of motility, and impaired events, such as acrosome reaction (74). Several studies have shown a significant reduction in oxidative stress or DNA damage after treatment with antioxidants (8). Indeed, the literature shows that antioxidants may play a primary role in protecting male germ cells against oxidative action (75). Our results encourage considering the alimentary approach of balanced food supplementation for the solution of dog subfertility. Balanced feed supplementation and integration could mitigate the negative impact of infertility in canine breeding.

## Conclusions

Food supplementation with PTHL can be considered an economic and natural method within an innovative multimodal approach to improve reproductive performances in canine breeding. The benefit of the supplemented diet with PTHL in enhancing semen quality and better supporting the antioxidant status of animals is an important goal for optimizing the male characteristics of reproductive efficiency. Some of the findings allow for speculation on the possibility of planning effect in subfertile and/or pathological male dogs. Therefore, the present article should set the base knowledge for further studies in male dogs with different physiological and pathological conditions.

## Data Availability Statement

The original contributions presented in the study are included in the article/Supplementary Material, further inquiries can be directed to the corresponding author/s.

## Ethics Statement

The animal study was reviewed and approved by Comitato Etico per la Sperimentazione Animale del Dipartimento di Medicina Veterinaria (CESA-DiMeV). Written informed consent was obtained from the owners for the participation of their animals in this study.

## Author Contributions

GA: investigation, formal analysis, writing—original draft, and writing—review and editing. VC: investigation and formal analysis. AM: conceptualization, investigation, data curation, formal analysis, writing—original draft, and writing—review and editing. MB and AB: investigation. AT: writing—review and editing. PD: conceptualization, investigation, funding acquisition, resources, project administration, and writing—review and editing. All authors contributed to the article and approved the submitted version.

## Funding

This research was funded by iGreen, Padua, Italy.

## Conflict of Interest

The authors declare that the research was conducted in the absence of any commercial or financial relationships that could be construed as a potential conflict of interest.

## Publisher's Note

All claims expressed in this article are solely those of the authors and do not necessarily represent those of their affiliated organizations, or those of the publisher, the editors and the reviewers. Any product that may be evaluated in this article, or claim that may be made by its manufacturer, is not guaranteed or endorsed by the publisher.
