# Supplementary material for: Effect of Pinus taeda Hydrolyzed Lignin on Biochemical Profile, Oxidative Status, and Semen Quality of Healthy Dogs
Source: Front Vet Sci. 2022 Jun 1;9:866112. doi: 10.3389/fvets.2022.866112 (PMC9198548; doi:10.3389/fvets.2022.866112)
Supplement: Supplementary file 1 [file Data_Sheet_1.docx]

|  | BREED | AGE | WEIGHT (Kg) | BCS _T -0_ | BCS _T 90_ |
| --- | --- | --- | --- | --- | --- |
| Athos | Mixed Breed | 4 | 23 | 3.5 | 3.5 |
| Igor | Corso | 3 | 30 | 3 | 3 |
| Kuma | Akita Inu | 5 | 27 | 3 | 3 |
| Max | Mixed Breed | 4 | 24 | 3 | 3.5 |
| Artù | Weimaraner | 3 | 26 | 3 | 3 |
| Porto | German shepherd | 5 | 29 | 3 | 3 |
| Quantum | German shepherd | 3 | 28 | 3 | 3 |
| Back | Weimaraner | 3 | 26 | 3 | 3 |
| Apollo | German shepherd | 4 | 27 | 3 | 3 |
| Rex | German shepherd | 3 | 30 | 3 | 3 |
| Mimmo | Mixed breed | 5 | 26 | 3 | 3 |
| Joy | Mixed breed | 4 | 27 | 3 | 3 |
| Spike | Corso | 3 | 28 | 3 | 3 |
| Ugo | Belgian malinois | 3 | 27 | 3 | 3 |
| Victor | German shepherd | 4 | 28 | 3 | 3 |
| Daniel | Mixed breed | 3 | 25 | 3 | 3 |
| Cesare | Mixed breed | 5 | 24 | 3 | 3 |
| Adam | Mixed breed | 3 | 26 | 3.5 | 3.5 |
| Gennaro | Mixed breed | 3 | 25 | 3 | 3 |
| Raja | Mixed Breed | 5 | 30 | 3.5 | 3.5 |

Tab.1 PTHL group

| Name | BREED | AGE | WEIGHT (Kg) | BCS _T -0_ | BCS _T 90_ |
| --- | --- | --- | --- | --- | --- |
| Ares | Mixed Breed | 4 | 25 | 3 | 3 |
| Pippo | Corso | 3 | 30 | 3 | 3 |
| Jonh | Mixed Breed | 5 | 27 | 3 | 3.5 |
| Lillo | Mixed Breed | 4 | 24 | 3 | 3 |
| Pegaso | German shepherd | 3 | 26 | 3 | 3 |
| Roky | Meticcio | 5 | 29 | 3 | 3 |
| Homer | German shepherd | 3 | 28 | 3 | 3 |
| Robert | Mixed Breed | 3 | 26 | 3 | 3.5 |
| Rio | Golden R. | 4 | 27 | 3 | 3 |
| Gigio | Mixed Breed | 5 | 30 | 3.5 | 3 |
| Roy | Mixed breed | 3 | 26 | 3 | 3 |
| rustin | Mixed breed | 4 | 26 | 3 | 3 |
| Jolly | Mixed breed | 3 | 26 | 3 | 3 |
| Libero | Mixed breed | 5 | 25 | 3 | 3 |
| Sheldon | Mixed breed | 3 | 27 | 3 | 3 |
| Kevin | Mixed breed | 4 | 29 | 3 | 3 |
| Vito | Belgian malinois | 4 | 28 | 3 | 3 |
| Splendor | Corso | 3 | 29 | 3 | 3 |
| Niky | Mixed breed | 3 | 25 | 3 | 3 |
| Zeus | Corso | 5 | 30 | 3 | 3 |

Tab.2 Control group
